# Prostatic Malakoplakia as a Mimic of Prostate Cancer Progression

**DOI:** 10.7759/cureus.91477

**Published:** 2025-09-02

**Authors:** Michael Spiker, Jacob D Pilley, Neel K Vaidya, Reza Manesh

**Affiliations:** 1 Internal Medicine, Veterans Affairs (VA) Greater Los Angeles, University of California, Los Angeles (UCLA), Los Angeles, USA; 2 Pathology, Veterans Affairs (VA) Greater Los Angeles, Los Angeles, USA; 3 Radiology, Veterans Affairs (VA) Greater Los Angeles, Los Angeles, USA

**Keywords:** anti-androgen therapy, case report, malakoplakia, prostate cancer, small-cell neuroendocrine carcinoma

## Abstract

Prostatic malakoplakia is a rare chronic inflammatory condition that can present as a cancer mimic and can co-occur with prostate adenocarcinoma. Typically, patients with prostate adenocarcinoma who present with evidence of disease progression while on androgen deprivation therapy (ADT) and a low prostate-specific antigen (PSA) level raise concern for transformation to small cell neuroendocrine carcinoma (SCNEC) of the prostate. A 67-year-old man with known locally advanced prostate adenocarcinoma was admitted with functional decline, radiographic tumor growth, and a low PSA. A repeat prostate biopsy was obtained to assess for transformation to SCNEC, but instead showed concurrent malakoplakia with his stable adenocarcinoma. With antibiotics, his peri-prostatic mass decreased in size, and he experienced an improvement in his functional status, allowing him to resume his ADT. Recurrent urinary tract infections, solid tumors, and immunosuppression are all risk factors for the development of prostatic malakoplakia, which is diagnosed pathologically from biopsy or after prostatectomy. Treatment is either long-term antibiotics or surgical resection. Prostatic malakoplakia should be considered as a mimic of disease progression in patients who have evidence of cancer progression despite a lower-than-expected PSA.

## Introduction

Patients with prostate adenocarcinoma who present with signs of disease progression despite suppressed levels of serum testosterone on androgen deprivation therapy (ADT) are diagnosed with castrate-resistant prostate cancer (CRPC). CRPC is diagnosed either by radiographic cancer progression or by serologic evaluation with increasing levels of prostate-specific antigen (PSA) [1]. When patients have radiographic evidence of disease progression with low PSA levels, it raises concern for transformation to small cell neuroendocrine carcinoma (SCNEC) of the prostate [2]. We present the case of a patient who was suspected of having a transformation to SCNEC of the prostate given his low PSA, functional decline, and radiographic suspicion of cancer progression but was surprisingly diagnosed with prostatic malakoplakia concurrently with his known prostate adenocarcinoma. This rare chronic inflammatory condition can manifest as a cancer mimic and present challenges in cancer staging for patients with known prostatic adenocarcinoma.

## Case presentation

A 67-year-old man with a history of prostate adenocarcinoma and opioid use disorder presented to the emergency department after nine months of recurrent painless hematuria, 40 pounds of weight loss over three months, and one month of progressive generalized weakness.

The patient’s hematuria had been intermittent and progressive over the nine months before admission, initially painless and without associated fevers. Subsequent episodes of gross hematuria worsened and were occurring almost daily, with intermittent fevers and suprapubic pain. He did not have night sweats.

The patient’s perceived weight loss was most dramatic over the month before admission, as he noticed that his clothes were too loose. He had minimal appetite and worsening abdominal cramping for the past three weeks that was not associated with oral intake. He did not have dysphagia or diarrhea. His mood was normal.

**Figure 1:**
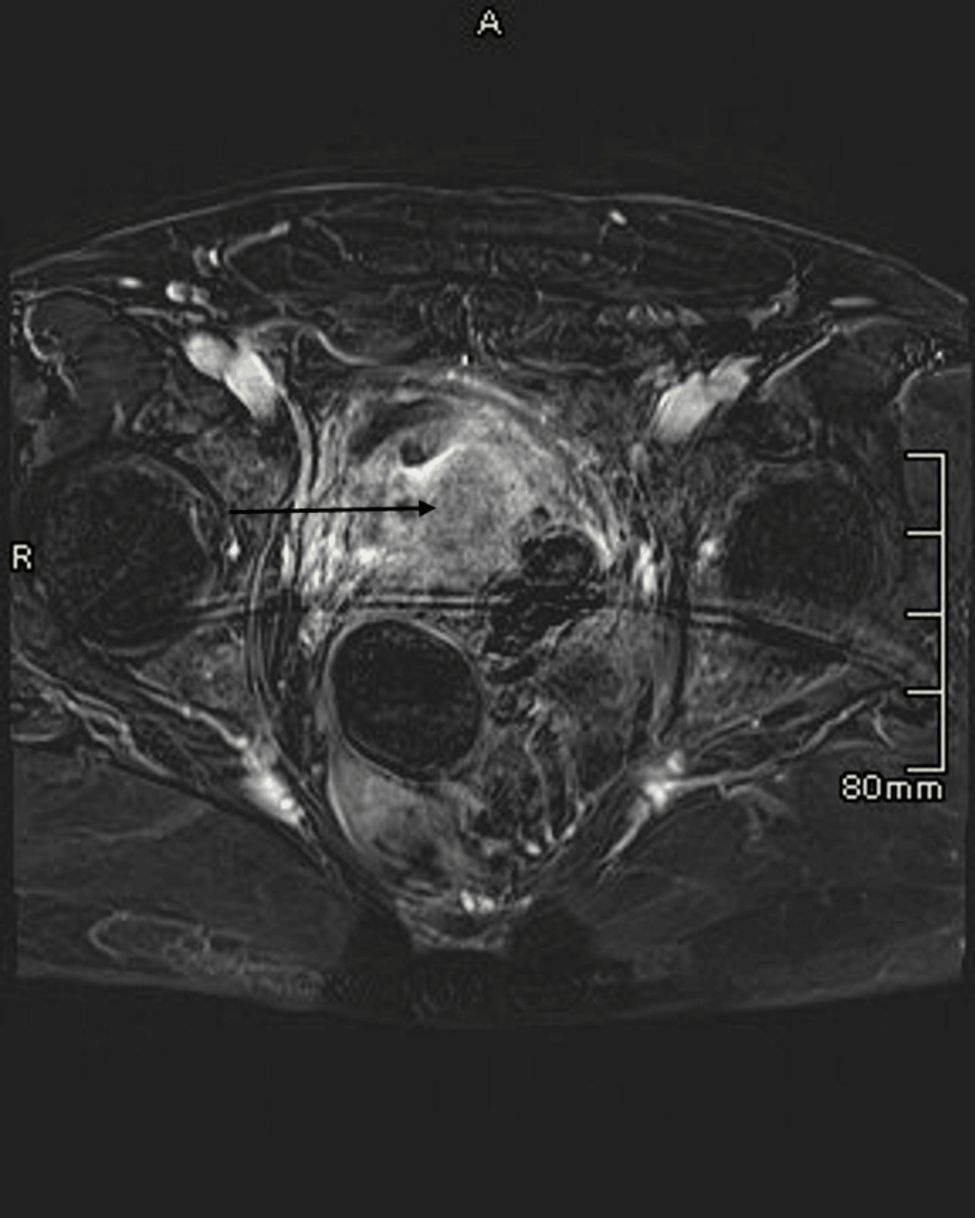
Prostate MRI Multiple non-enhancing T1 hyperintense, heterogeneous T2 signal intensity cystic lesions in the pelvis. The lesions appear to arise from the posterior wall of the prostate and exert a mass effect on the left anterolateral wall of the rectum. No definite communication with the rectal lumen. MRI: magnetic resonance imaging

Six months prior to admission, he was well-appearing, with a weight of 170 lb, stable vital signs, and laboratory results notable for a hemoglobin level of 10.1 g/dL (reference value: 13.8-17.2 g/dL) and a creatinine level of 1.1 mg/dL (reference value: 0.74-1.35 mg/dL). To assess his hematuria, he received a CT urogram followed by an MRI of his pelvis showing a cystic mass adjacent to the prostate and rectum (6.4 x 5 cm) and enlarged left pelvic lymph nodes (Figure 1).

A follow-up colonoscopy showed no luminal mass, although there was luminal narrowing from extrinsic compression of the peri-prostatic cystic mass. Interventional radiology-guided aspiration of the cystic mass demonstrated atypical cells, with subsequent left iliac lymph node biopsy showing nodal evidence of adenocarcinoma, favoring prostatic origin. A follow-up prostate core needle biopsy showed adenocarcinoma with a Gleason score of 4+4. PSA at the time was 13 ng/mL (normal: <1.5 ng/mL).

Five months prior to admission, he was treated with antibiotics as an outpatient for two separate urinary tract infections (*Enterococcus faecalis* and *Pseudomonas aeruginosa*). Four months before admission, he was hospitalized for sepsis secondary to an *Escherichia coli* urinary tract infection and bacteremia. After discharge, he was started on enzalutamide (an androgen receptor signaling inhibitor) and degarelix (a gonadotropin-releasing hormone antagonist).

The patient continued enzalutamide and had scheduled follow-up with oncology and urology. Three months before admission, he had relapsed on fentanyl and missed additional follow-up appointments.

On admission, he was afebrile and normotensive, with a normal heart rate, weighing 130 lb, down from 170 lb six months prior. He was a chronically ill-looking middle-aged man, uncomfortably lying in bed. He had bitemporal wasting. Cardiac and pulmonary exams were normal. He had a mildly distended, nontender abdomen with dullness to percussion and a positive fluid wave. He had a stage IV sacral wound without purulence or surrounding erythema and symmetric mild pitting edema of his lower extremities. The rest of his exam was normal.

Initial laboratory assessment demonstrated a hemoglobin of 7.5 g/dL (reference value: 13.8-17.2 g/dL, from a prior baseline of 10 g/dL), WBC 14.7 x 103 μL (reference value: 4 x 103-11 x 103 μL) that was neutrophil predominant, and creatinine of 3.8 mg/dL (reference value: 0.74-1.35 mg/dL, baseline of 1.0 mg/dL). His previously elevated PSA was now undetectable, and his liver enzymes were within normal limits. The urinalysis revealed 3+ WBC, was nitrite-positive, and was leukocyte esterase-positive.

**Figure 2:**
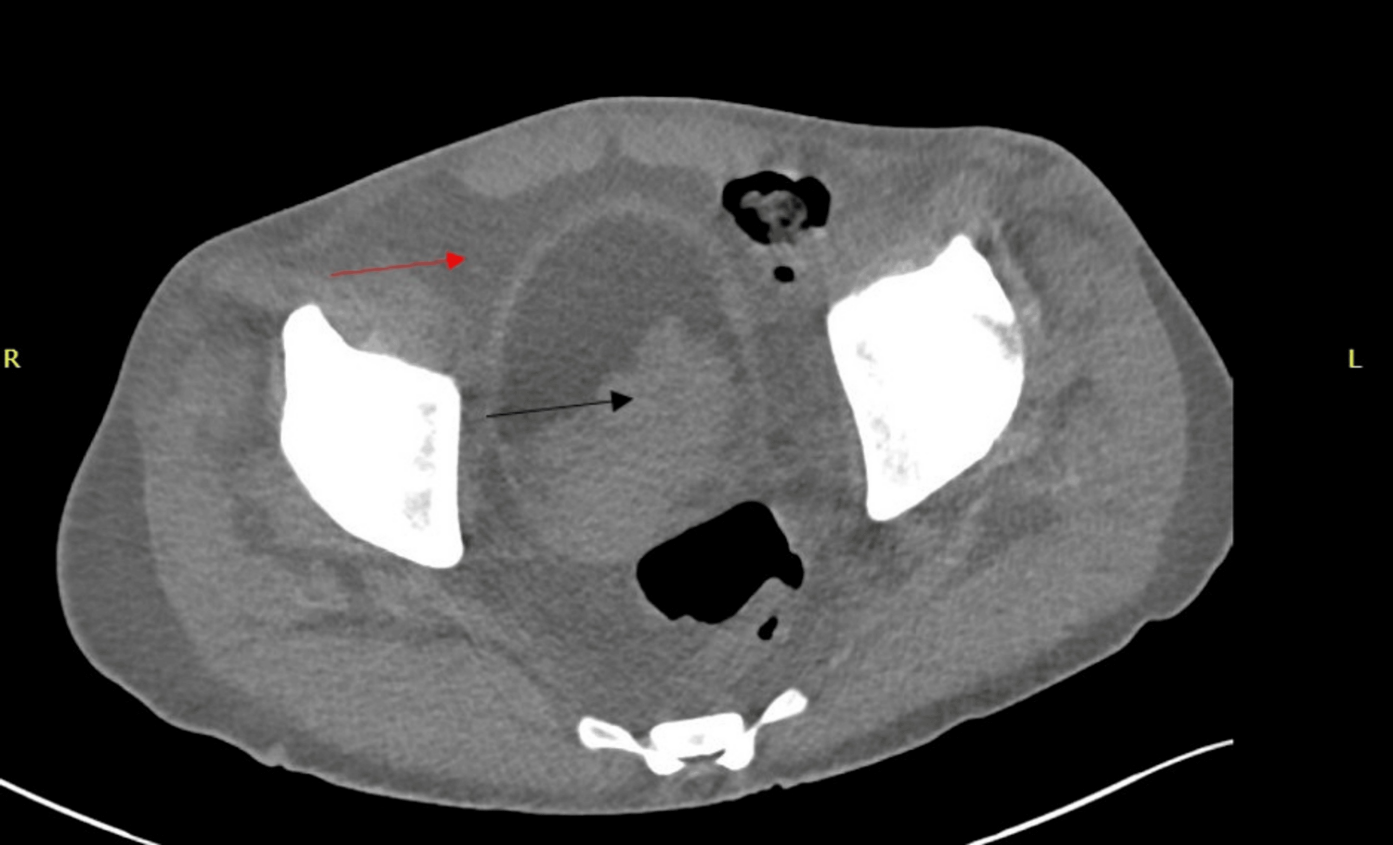
Non-contrast CT abdomen and pelvis Severe bilateral hydronephrosis and hydroureter to the level of the pelvic mass. Interval increase in size of the mass (black arrow) in the region of the prostate compatible with disease progression. The cystic component of the mass is poorly evaluated secondary to limitations of the study. Moderate ascites (red arrow). CT: computed tomography

A non-contrast CT abdomen and pelvis showed growth of the previously seen peri-prostatic cystic mass (now 8.8 x 7.7 cm; had been 6.4 x 5.6 cm), enlarging pelvic adenopathy, bilateral hydronephrosis from the enlarging peri-prostatic mass, and new ascites (Figure 2).

Admission blood cultures and urine cultures grew *Escherichia coli*, and a diagnostic paracentesis revealed a transudative fluid without signs of infection or malignancy. The repeat left iliac node core biopsy was negative for malignancy.

**Figure 3:**
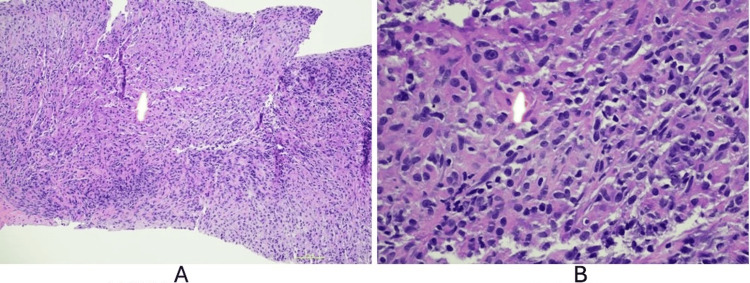
Prostate biopsy H&E stain (A) H&E 100x showing sheets of foamy epithelioid histiocytes with granular eosinophilic cytoplasm. (B) H&E 400x showing Michaelis-Gutmann bodies in a high-power image, consistent with classic malakoplakia. H&E: hematoxylin and eosin

For his obstructive hydronephrosis, he received urgent percutaneous nephrostomy tubes with improvement in his kidney function. He was treated with 10 days of IV ceftriaxone to treat *Escherichia coli* bacteremia secondary to a urinary tract infection. With radiographic evidence of disease progression and a low PSA, he had a repeat prostate biopsy out of concern for SCNEC transformation of his prostate cancer. This concern existed because adenocarcinoma secretes PSA, and prostatic SCNEC is a neuroendocrine tumor that does not secrete PSA. A repeat prostate biopsy instead showed sheets of foamy epithelioid histiocytes with granular eosinophilic cytoplasm and Michaelis-Gutmann bodies consistent with malakoplakia in addition to his known prostate adenocarcinoma (Figure 3).

**Figure 4:**
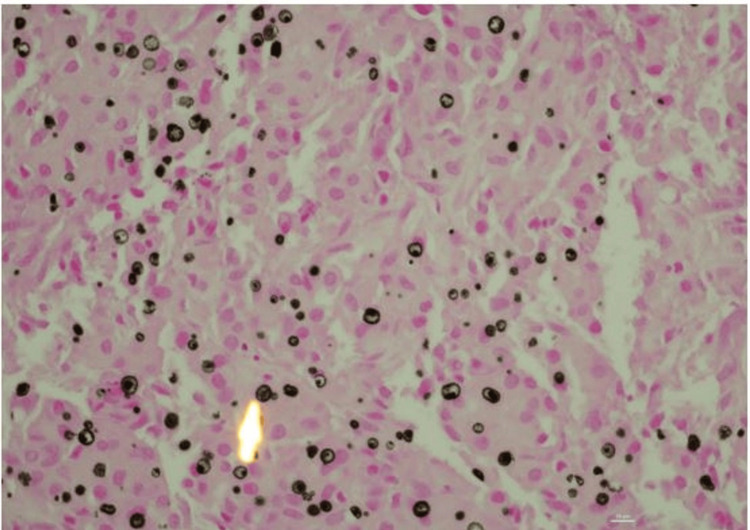
Prostate biopsy von Kossa stain The Von Kossa stain highlights Michaelis-Gutmann bodies.

The ongoing presence of some adenocarcinoma in his prostate (Gleason 4+4), even with a depressed PSA, was expected given that he had only been on treatment for a few months and had locally advanced disease at diagnosis. Von Kossa staining (a calcium stain that more explicitly identifies Michaelis-Gutmann bodies) also highlighted Michaelis-Gutmann bodies, pathognomonic for malakoplakia (Figure 4) [3-5].

Given the patient’s poor functional status on admission, his malakoplakia was treated with a three-month course of levofloxacin instead of surgical resection. Levofloxacin was chosen because it acts intracellularly and targets his prior cultured urinary pathogens as part of a treatment plan for his malakoplakia. After starting antibiotics, he had significant functional improvement and later left the hospital ambulatory, with a resolving pressure wound and 20 pounds of weight gain over six weeks.

**Figure 5:**
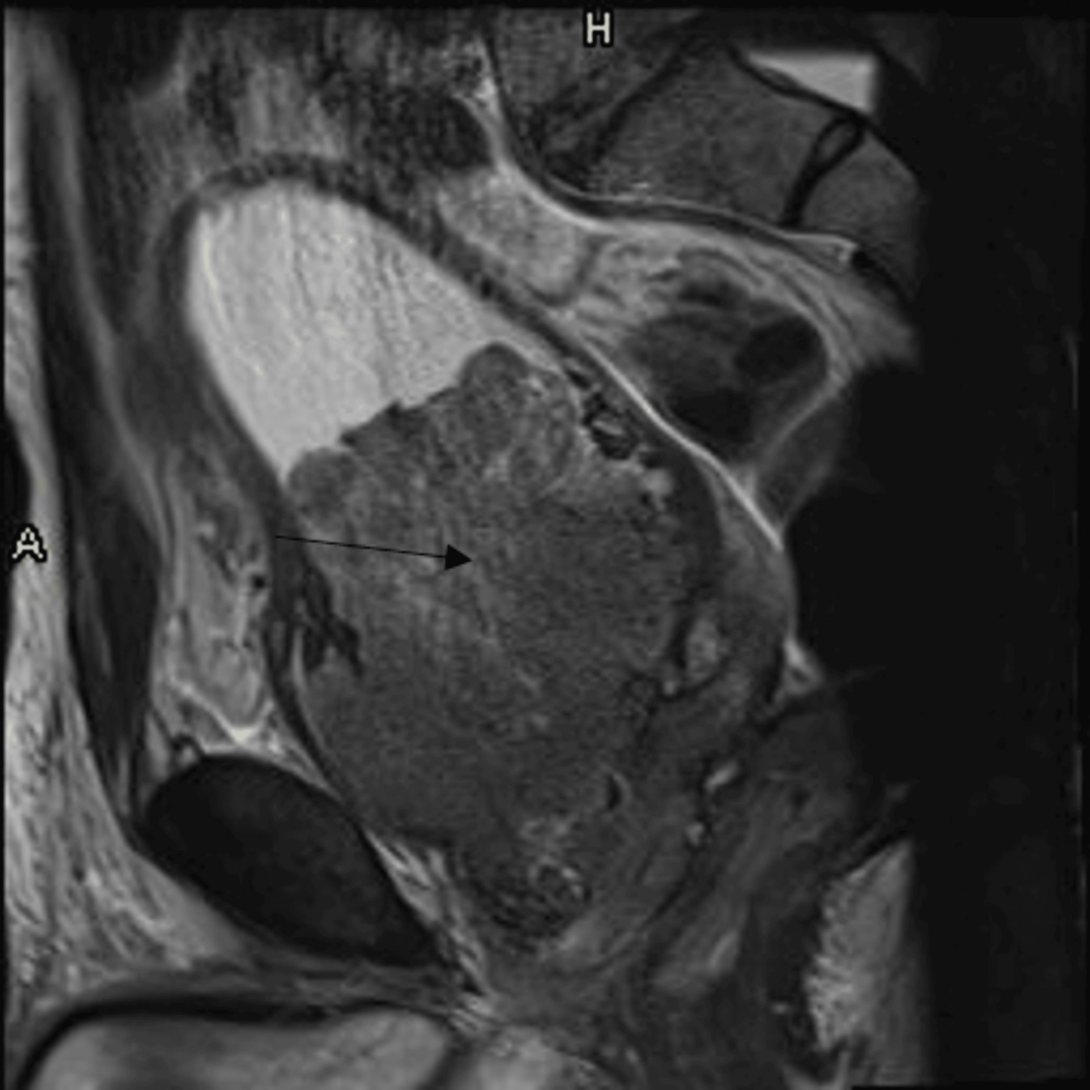
Prostate MRI The previously seen multiple non-enhancing cystic lesions in the pelvis have completely resolved in the interval. MRI: magnetic resonance imaging

After discharge, he continued to show ongoing improvement in his weight gain, functional status, and lower urinary tract symptoms. A follow-up MRI of his prostate two months after discharge demonstrated a near resolution of the cystic component of his prostatic mass (thought to be from treatment of his malakoplakia) (Figure 5).

However, he had progressive enlargement of the prostate gland with signs of invasion into the bladder, which was likely a progression of his adenocarcinoma. He continued to follow up with urology, infectious disease, and medical oncology. Unfortunately, nine months after discharge, he developed bone metastases and an increasing PSA (up to 37 ng/mL) and was diagnosed with CRPC. He eventually died comfortably in an inpatient hospice with family present after a complex hospitalization three months later.

## Discussion

For patients presenting with known advanced prostate adenocarcinoma and radiographic evidence of disease progression while on ADT and a low PSA, initial consideration is made for co-occurring SCNEC. Clinically, SCNEC of the prostate can present aggressively, often with visceral disease and a low PSA level despite having a significant metastatic burden. SCNEC of the prostate is not responsive to hormone therapy and is more often found co-occurring with adenocarcinoma than as an isolated finding [2].

Identifying malakoplakia as the cause of the increased size of the patient’s peri-prostatic mass and not progression or transformation of his cancer was critical in appropriately treating and prognosticating him. In most solid cancer subtypes, when patients become bedbound with advanced pressure injuries, they have a prognosis of less than six months and are not candidates for systemic cancer therapy [6]. Without a repeat prostate biopsy, that assumption could have been made in this patient. Instead, reframing his clinical decline as being secondary to sequelae of his malakoplakia helped him receive appropriate antibiotic therapy for a treatable condition and eventually restart ADT for his known prostate adenocarcinoma. Without this information, he would have been inaccurately assumed to be prognostically appropriate for hospice.

In this patient, the interval enlargement of his known peri-prostatic mass was secondary to malakoplakia, a rare chronic inflammatory disease first diagnosed on a bladder biopsy by Michaelis and Gutmann in 1902. Michaelis and Gutmann identified calcified intracytoplasmic inclusions (subsequently named Michaelis-Gutmann bodies), thought to represent partially degraded bacterial organisms. Malakoplakia is thought to be caused by an acquired bactericidal defect in macrophages, with gram-negative bacteria, especially *Escherichia coli*, most often implicated. It is typically observed within the urinary tract but has also been detected in almost every organ system. Risk factors can be acquired immunodeficiencies, including post-transplant status, solid malignancy, diabetes, and HIV [4,5,7].

Prostatic malakoplakia is a rare clinical entity, with the largest case series including 49 patients. The median age was 67, and the PSA level measured was only moderately elevated, with an average PSA of 7.5 ng/mL. Prostate adenocarcinoma is also often found co-occurring on biopsy, appearing in 37% of patients in this case series. It remains unclear why solid cancers are a risk factor for malakoplakia. Still, it has been hypothesized for prostate cancer that this could be temporally associated with needle biopsy, especially in patients with recurrent urinary tract infections.

Additionally, it has been postulated that the local immunomodulatory effects of cancer increase the risk of malakoplakia. For example, 40% of patients with gastrointestinal malakoplakia had also been diagnosed with colon or rectal cancer [3]. For patients with positive urine cultures, *Escherichia coli* was the most commonly isolated pathogen. Often, patients experience chronic lower urinary tract symptoms that precede the diagnosis, with nodules often felt during a digital rectal exam [4,5]. The diagnosis is typically made pathologically, either on core biopsy or after radical prostatectomy [4]. When histologic features are suggestive of malakoplakia (granulomatous inflammation with intracytoplasmic, sharply demarcated, concentric, and basophilic inclusions), staining is performed to confirm the diagnosis. The inclusions will stain black with Von Kossa and magenta with PAS-D, which confirms that the intracytoplasmic inclusions are Michaelis-Gutmann bodies, a diagnostic feature of malakoplakia [4,5].

In this case, the patient had multiple risk factors for prostatic malakoplakia, including prior urinary tract infections with gram-negative pathogens, active prostate adenocarcinoma, and prostate biopsy [4,5]. In patients where malakoplakia co-occurs with prostate adenocarcinoma, it is typically found incidentally on prostate biopsy or prostatectomy. This case was unusual because it mimicked cancer progression, and the patient clinically improved with antibiotics alone. Given his other risk factors, it is difficult to know if his prostate biopsy could have caused his malakoplakia or if it preceded the biopsy but was not seen because of sampling error. Treatment of prostate malakoplakia includes either a prolonged course of antibiotics that act intracellularly or surgical resection [4,5].

## Conclusions

Prostate malakoplakia is a rare clinical entity that can occur with recurrent urinary tract infections and can be found with prostate adenocarcinoma, leading to challenges in appropriate staging and treatment. Although it can present as a cancer mimic given its ability to form nodules or masses, it is crucial to add it to the differential diagnosis of conversion to SCNEC when encountering an enlarging prostate mass in someone with known prostate adenocarcinoma and a down-trending PSA. For patients with known prostate adenocarcinoma, if the PSA is only moderately elevated despite more locally advanced evidence of malignancy radiographically, it is reasonable to convey suspicious findings to the pathologist so that malakoplakia can be excluded, improving the diagnostic sensitivity of this cancer mimic.

## References

[1] Vellky JE, Ricke WA (2020). Development and prevalence of castration-resistant prostate cancer subtypes. Neoplasia.

[2] Nadal R, Schweizer M, Kryvenko ON, Epstein JI, Eisenberger MA (2014). Small cell carcinoma of the prostate. Nat Rev Urol.

[3] Medlicott S, Magi-Galluzzi C, Jimenez RE, Trpkov K (2016). Malakoplakia associated with prostatic adenocarcinoma: report of 4 cases and literature review. Ann Diagn Pathol.

[4] Acosta AM, Sangoi AR, Maclean F (2022). Prostatic malakoplakia: clinicopathological assessment of a multi-institutional series of 49 patients. Histopathology.

[5] Ho M, Wu J, Skinnider B, Kavanagh A (2018). Prostatic malakoplakia: a case report with review of the literature. J Surg Case Rep.

[6] Jang RW, Caraiscos VB, Swami N (2014). Simple prognostic model for patients with advanced cancer based on performance status. J Oncol Pract.

[7] Bagnasco S, Gautam SC (2024). Renal malakoplakia. N Engl J Med.

